# Histamine release and fibrinogen adsorption mediate acute inflammatory responses to biomaterial implants in humans

**DOI:** 10.1186/1479-5876-5-31

**Published:** 2007-07-01

**Authors:** Johann Zdolsek, John W Eaton, Liping Tang

**Affiliations:** 1 Department of Hand and Plastic Surgery, University Hospital, SE-581 85 Linköping, Sweden; 2 Molecular Targets Program, Brown Cancer Center, University of Louisville, Louisville, KY 40202, USA; 3 Bioengineering Department, University of Texas at Arlington, Arlington, TX 76019, USA

## Abstract

**Background:**

Medical implants often fail as a result of so-called foreign body reactions during which inflammatory cells are recruited to implant surfaces. Despite the clinical importance of this phenomenon, the mechanisms involved in these reactions to biomedical implants in humans are not well understood. The results from animal studies suggest that both fibrinogen adsorption to the implant surface and histamine release by local mast cells are involved in biomaterial-mediated acute inflammatory responses. The purpose of this study was to test this hypothesis in humans.

**Methods:**

Thirteen male medical student volunteers (Caucasian, 21–30 years of age) were employed for this study. To assess the importance of fibrinogen adsorption, six volunteers were implanted with polyethylene teraphthalate disks pre-coated with their own (fibrinogen-containing) plasma or (fibrinogen-free) serum. To evaluate the importance of histamine, seven volunteers were implanted with uncoated disks with or without prior oral administration of histamine receptor antagonists. The acute inflammatory response was estimated 24 hours later by measuring the activities of implant-associated phagocyte-specific enzymes.

**Results:**

Plasma coated implants accumulated significantly more phagocytes than did serum coated implants and the recruited cells were predominantly macrophage/monocytes. Administration of both H1 and H2 histamine receptor antagonists greatly reduced the recruitment of macrophages/monocytes and neutrophils on implant surfaces.

**Conclusion:**

In humans – as in rodents – biomaterial-mediated inflammatory responses involve at least two crucial events: histamine-mediated phagocyte recruitment and phagocyte accumulation on implant surfaces engendered by spontaneously adsorbed host fibrinogen. Based on these results, we conclude that reducing fibrinogen:surface interactions should enhance biocompatibility and that administration of histamine receptor antagonists prior to, and shortly after, medical device implantation should improve the functionality and longevity of medical implants.

## Background

Implanted devices are increasingly important in the practice of medicine. In an NIH news release dated January 5^th ^2000, it was estimated that 8 to 10 percent of the U. S. population currently have permanent medical implants [1]. Although most implant materials are inert, non-immunogenic and non-toxic, devices made of such materials often trigger a variety of adverse reactions. These include surface-mediated thrombosis associated with blood contact surfaces [2], complement activation induced by haemodialysis membranes [3], inflammation surrounding many types of implants [4], device-centered infections [5] and fibrotic tissue formation around tissue implants and prostheses [6]. These complications may cause the failure of many types of medical implants, often requiring surgical removal and replacement, increasing both the risk to patients and the cost of health care. Consequently, intensive research efforts have been devoted to the development of novel strategies to improve tissue compatibility of medical devices. However, improvements in biocompatibility have been hindered by our lack of understanding of the basic mechanisms involved in human tissue responses to biomaterial implants.

Because biomaterials spontaneously accumulate a layer of adsorbed plasma proteins prior to inflammatory cell accumulation, it is widely accepted that the types and species of adsorbed proteins play an important role in the pathogenesis of biomaterial-mediated acute inflammatory responses. Using an animal implantation model, we earlier found that spontaneously adsorbed (and partially denatured) fibrinogen is a critical mediator of acute inflammatory responses to biomaterial implants [7]. In support of this, we observed much less phagocyte accumulation on the surfaces of serum-coated implants than on implants coated with plasma. As a more direct test, hypo-fibrinogenemic mice were generated with repeated ancrod injection. Biomaterial implants in these mice failed to accumulate adherent phagocytes (unless the implants were pre-coated with murine fibrinogen) [7]. We subsequently found that, following initial adsorption on hydrophobic biomaterial surfaces, fibrinogen undergoes conformational changes which expose previously occult epitopes on the gamma chain of fibrinogen ('P1' (γ190–202) and 'P2' (γ377–392)) [8,9]. These newly exposed epitopes are responsible for triggering the recruitment and activation of phagocytes, early events in the cascade of events involved in foreign body reactions.

In mice, the initial recruitment of inflammatory cells to experimental implants is mediated by histamine. Mast cell deficient mice showed greatly diminished phagocyte accumulation on implants and administration of H1 and H2 receptor antagonists to normal mice substantially reduced phagocyte recruitment [10].

Despite these earlier observations, it has remained uncertain whether adsorbed fibrinogen and histamine release play similar roles in triggering foreign body reactions in humans. The goal of this investigation was to determine whether these earlier results in animal models were directly pertinent to humans given experimental biomaterial implants.

## Methods

### Test materials and chemicals

Polyethylene terephthalate (PET) film, type A, 0.005 mm thick, was obtained from Cadillac Plastic and Chemical (Birmingham, MI, USA). All other reagents were purchased from Sigma Chemical Co. (St Louis, MO, USA).

### Preparation of uncoated and protein-coated PET disks

The PET film was cut into circular disks of 12 mm diameter with punch and die set (Precision, Downers Grove, IL). The punch and die set was sharpened prior to cutting to ensure smooth edge. The disks were first cleaned with multiple changes of 100% ethanol to remove the residual fibers and debris. The disks were then washed with several changes of 70% ethanol and then autoclaved at 125°C for 15 minutes. To test the importance of surface fibrinogen in the aetiology of biomaterial-mediated inflammatory responses, 20 ml of venous blood from each volunteer was drawn into collection tubes with and without anticoagulant (sodium heparin). The heparinized blood tubes were centrifuged at 200 × g for 10 minutes to produce plasma. Clot tubes without anticoagulant were cooled to 4°C for 120 min to allow for blood coagulation and then centrifuged to produce sera. The PET disks were incubated in plasma or serum from each volunteer overnight (12 h) under sterile conditions in sealed vials in an incubator at 37°C. The protein-coated disks were thoroughly washed in sterile phosphate buffered saline (PBS) before implantation.

### Human volunteers

We recruited 13 healthy male Caucasian medical student volunteers aged 21–30 years. They were divided into two groups where 6 (average age = 24.5 years) took part in experiments on the effects of histamine receptor antagonists and 7 (average age = 23.8 years) were involved in studies of the effects of fibrinogen. None of the volunteers had taken any medication for two weeks prior to the experiments. These studies were approved by the Human Ethics Committee of the Linköping University Hospital. Following written and oral instruction, all volunteers gave written consent to their participation.

### Experimental setup

#### Effects of fibrinogen (n = 7)

Day 1: Withdrawal of blood to obtain plasma and sera. PET disks were incubated with plasma and sera for 12 hours.

Day 2: Implantation of an autologous plasma coated disk in the left arm and an autologous serum coated disk in the right arm of all 7 volunteers.

Day 3: Explantation of both disks after 24 hours.

#### Antihistamine treatment (n = 6)

Day 1: Implantation of an uncoated disk in the right arm.

Day 2: Explantation of the disk after 24 hours. The same patients were immediately given 10 mg of the H1 histamine receptor antagonist Cetirizine and 20 mg of the H2 histamine receptor antagonist Famotidine, both *per os*. Two hours later, a new uncoated disk was implanted in the left arm. The administration of Famotidine was repeated 12 hours after the first dose to ensure an adequate plasma level of the drug and continuous histamine receptor blockade.

Day 3: Explantation of the second disk after 24 hours implantation.

### Surgical procedure for biomaterial implantation

After sterile preparation and disinfection of the skin on the inside of the upper arm with 0.02% chlorhexidine and 70% ethanol, local anaesthesia was performed using 1 ml lidocaine (10 mg/ml). A transverse 15 mm long incision was made 5 min after injection of the anaesthetic and a small subcutaneous pocket formed. A sterile PET disk, either coated or uncoated, was then placed in the pocket and the skin was closed using sterile tape (Steristrips^®^, 3 M). Explantation was performed 24 h after implantation as follows: the wound-tape was removed, the wound edges separated and the disks removed with a forceps for later analyses. The skin was then closed with sterile tape. The 24 hour time period was chosen because our earlier investigations in mice indicated that in a subcutaneous implantation model this was the time of maximal accumulation of PMN and MØ. A pilot study was also carried out in 2 human volunteers (in addition to the 13 human volunteers mentioned above). The results from this limited preliminary human trial indicated that 24 hour-implantation is appropriate to assess acute inflammatory responses to biomaterial implants in humans. However, we should note that it is not presently known whether this represents the time of maximal phagocyte accumulation.

Throughout the study, no voluteer had prominent signs of inflammatory reactions and erythema in or around the implantation sites. Because of the localized and minimal inflammatory responses, it is unlikely that the tissue responses to the first implants would affect responses to the second implants in the same volunteers.

### Analyses of biomaterial-mediated inflammatory responses

Immediately following explantation, the PET disks were washed with isotonic PBS. To reduce the edge effect on the quantification of implant-associated inflammatory responses, the edges of all explanted disks were cleaned by sliding the edges of the disks across tissue paper to dislodge cells adherent to the implant edge. The disks were then placed in 0.6 ml of 1% (vol/vol) Triton X-100 for 1 hour to release cytoplasmic and granular enzymes from implant-associated cells [7-10]. Because biomaterial-mediated inflammatory responses are reflected by the accumulation of phagocytes on implant surfaces, the number of adherent phagocytes was taken as a measure of the extent of foreign body reactions. Implant-associated myeloperoxidase (MPO) and non-specific esterase (NSE) were quantified to assess the numbers of adherent polymorphonuclear neutrophils [PMN] and macrophages/monocytes [MØ], respectively.

MPO was measured by the guaiacol reaction [7-10] in the presence of 1 mM (final concentration) 3-amino-1,2,4-triazole to inhibit eosinophil peroxidase [11]. Total MPO activity was taken as a measure of surface associated PMN. Control studies on purified PMN from human venous blood indicated that the MPO activity of human peripheral PMN is about 45 nU/cell.

Non-specific esterase (NSE), a cytoplasmic enzyme, resides specifically in monocytes/macrophages [12] and was used to assess the numbers of adherent MØ [7-10]. NSE activity was determined by measuring the rate of hydrolysis of *p*-nitrophenyl butyrate [13] in the presence of a cholinesterase inhibitor, eserine (10 mM, final concentration) [14]. Control studies on human peritoneal macrophages (recovered from peritoneal lavage fluids from patients being given chronic ambulatory peritoneal dialysis) indicated that the NSE activities of human MØ are about 20 nU/cell.

### Statistical and power analyses

As the results were normally distributed, paired Student's t-test was used. A p-value of <0.05 was considered significant. The power analyses of the number of test subjects were carried out using G* power 3 program [15].

## Results

### Importance of fibrinogen adsorption in implant-mediated acute inflammatory response in humans

**Figure 1:**
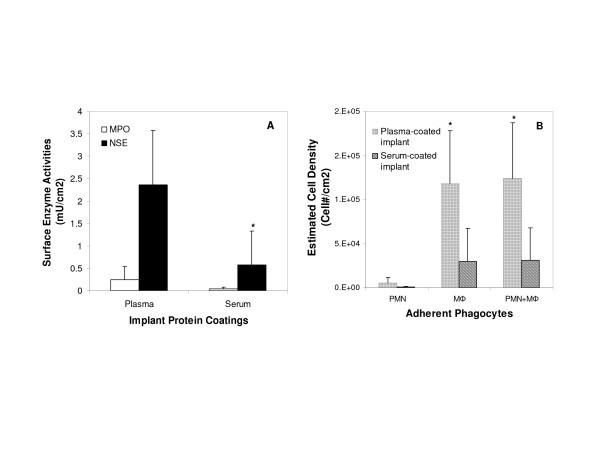
Phagocyte accumulation on the surfaces of PET disks implanted in human volunteers, precoated with either plasma or serum derived from each volunteer. (A) Myeloperoxidase and non-specific esterase activities were measured on disks explanted after 24 h to reflect the degree of accumulation of PMN and MØ, respectively. (B) The estimated numbers of adherent PMN, MØ, and total phagocytes (PMN+ MØ) were calculated. Vertical lines denote ± 1 Standard Deviation. Significance vs. plasma-coated disks: * p < 0.05.

To determine the importance of implant-bound fibrinogen in triggering biomaterial-mediated inflammatory responses in humans, PET disks precoated with the plasma or serum of each volunteer were implanted subcutaneously and then retrieved 24 hours later. As was true in the mouse model, plasma-coated surfaces accumulated many more PMN and MØ compared to serum-coated disks. The average numbers of adherent PMN and MØ on plasma-coated surfaces were 4–5 fold higher than those on serum-coated disks (Figure 1.A). Interestingly, and in contrast to mice, the majority (>95%) of implant-associated phagocytes were MØ (possibly a function of differences in the site of implantation – intraperitoneal in mice and subcutaneous in humans). Perhaps most importantly, the aggregate numbers of adherent phagocytes (PMN+MØ) on plasma coated surfaces were significantly higher than those on serum coated surfaces (Figure 1.B). Power analyses were carried out using G* power 3 and the results indicated that 7 volunteers recruited for this investigation were sufficient to achieve power = 0.8 and alpha = 0.05 in all comparisons (i.e., PMN, MØ and PMN + MØ numbers).

### Importance of histamine in implant-mediated inflammatory responses in mice and humans

**Figure 2:**
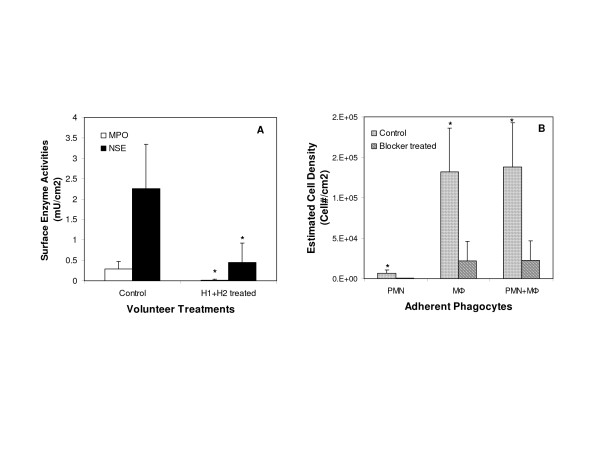
Phagocyte accumulation on the surfaces of PET disks implanted in volunteers with and without the treatment of H1 and H2 histamine receptor antagonists. (A) Myeloperoxidase and non-specific esterase activities were measured to reflect the degree of accumulation of PMN and MØ, respectively. (B) The estimated numbers of adherent PMN, MØ, and total phagocytes (PMN+ MØ) are calculated. Vertical lines denote ± 1 Standard Deviation. Significance vs. control volunteers (without antagonists): * p < 0.05.

In previous work with the mouse implantation model, we found that histamine receptor antagonists could be used to reduce biomaterial-mediated acute inflammatory responses [**Error! Bookmark not defined.**] and that the simultaneous administration of H1 and H2 histamine receptor antagonists greatly decreased the numbers of implant-associated PMN and MØ. As was true in mice, administration of a combination of H1 and H2 histamine receptor antagonists suppressed the accumulation of both PMN and MØ on subcutaneous implants in humans (Figure 2). The accumulation of PMN (p = 0.016), MØ (p = 0.011), and aggregate numbers of PMN + MØ (p = 0.011) was significantly less on uncoated PET disks implanted in volunteers treated with the histamine receptor antagonists compared with implants in the same volunteers without antihistamine treatment. G* 3 power analyses were also carried out in this case and revealed that 6 patients were sufficient to achieve power >0.8 and alpha = 0.05.

## Discussion

The implantation of medical devices often leads to foreign body reactions which are driven by the accumulation and activation of inflammatory cells. These acute inflammatory responses are very often followed by chronic inflammation and fibrosis [16]. These reactions have been linked to the degradation and failure of many types of implants, including pacemaker leads [17], mammary prostheses [18], temporomandibular [19] and other joint implants [20]. To improve the biocompatibility and safety of medical devices, intensive research efforts have been placed on the development of biomaterials with enhanced tissue compatibility. However, such efforts have been hindered by the lack of knowledge of the mechanisms governing foreign body reactions.

In an attempt to understand these basic mechanisms, we earlier carried out a series of experiments using a murine implantation model [4,7-10]. Our investigations revealed that, rapidly following implantation, biomaterial surfaces become covered with a layer of host proteins. We found that the adsorption and later "denaturation" of fibrinogen is the main factor in triggering biomaterial-mediated inflammatory responses in mice [7]. Furthermore, it appeared that histamine release by mast cells in the vicinity of the implant was important in facilitating the recruitment of inflammatory cells inasmuch as both mast cell deficient mice and mice treated with histamine receptor antagonists had greatly reduced acute inflammatory responses to implanted biomaterials.

However, it was not clear whether these processes, found to be important in mice, might be similarly crucial in humans with implanted biomaterials. As a first test of this, we have examined the importance of adsorbed fibrinogen in prompting foreign body reactions in human volunteers. As was true in the earlier murine model, plasma coated implants attracted significantly more phagocytes than did serum coated implants in humans. The process of inflammatory cell recruitment – in both mice and humans – likely involves the tendency of fibrinogen to adsorb and subsequently denature on the hydrophobic surfaces of biomedical polymers [7]. The "denaturation" of adsorbed fibrinogen is particularly important inasmuch as it leads to the exposure of two epitopes on the fibrinogen gamma chain (P1 and P2) which are normally occult in soluble fibrinogen. The exposure of these short sequences is required for both the adhesion and activation of phagocytes [8,9]. Indeed, the degree of P1/P2 exposure engendered by different types of biomedical polymers predicts the extent of biomaterial-mediated inflammatory responses [9]. Furthermore, it appears that the interaction between phagocytes and surface P1/P2 epitope is via the Mac-1 (CD11b/CD18) integrin, which is upregulated on inflammatory cells recruited to the site of the implant [21,22].

Our earlier animal studies also indicated that histamine, released from activated mast cells, is critical to the recruitment of phagocytes to both subcutaneous and intraperitoneal implants [10]. This is in accord with numerous observations that biomedical implants trigger both edematous and hyperemic responses typically mediated by histamine. The pro-inflammatory effects of the released histamine evidently involve both H1 and H2 receptors [10]. In the present experiments on humans, we observed that treatment with a combination of H1 and H2 receptor antagonists reduced by more than 80% the accumulation of phagocytes on implant surfaces as was also true in the murine models. Since histamine exerts its action on capillary permeability and phagocyte transmigration through endothelial barrier [23,24], it is likely that histamine receptor antagonists diminish initial phagocyte recruitment probably through suppression of implant-mediated hyperemia and loosening of the endothelial barrier. This suggests that histamine antagonist administration shortly before and after the placement of biomedical implants in humans may lessen the phagocyte-mediated foreign body responses and later reactions such as fibrotic capsule formation around implanted medical devices.

## Conclusion

To the best of our knowledge, this is the first experimental study on the molecular determinants of biomaterial-mediated acute inflammatory responses in humans. The results support the general conclusion that biocompatibility might be improved by the design of surfaces which reduce fibrinogen adsorption and denaturation. The incubation of medical devices with patient's serum may also reduce foreign body reactions to the implants. Furthermore, our results confirm the importance of histaminic responses in the pathogenesis of biomaterial-mediated inflammatory responses. These latter observations may have practical implications, in that treatment of implant recipients with histamine receptor antagonists could be used to limit both acute inflammatory responses and later fibrotic reactions (which may directly stem from the acute responses). Patients who might benefit from such antihistamine treatment include those being treated with joint implants, breast implants, tissue engineering implants and drug delivery devices.

## Competing interests

The author(s) declare that they have no competing interests.

## Authors' contributions

JZ conducted the human study. LT performed data analyses. JZ, JWE, LT co-wrote the manuscript. All authors read and approved the final manuscript.
